# Effectiveness of Early Physiotherapy Interventions with Guided Parental Involvement on Motor Development in Preterm Infants: A Systematic Review

**DOI:** 10.3390/pediatric18040100

**Published:** 2026-07-29

**Authors:** Georgios Grammatikou, Efterpi Pavlidou, Nikolaos Strimpakos, Konstantinos Chandolias

**Affiliations:** 1 Health Assessment and Quality of Life Research Lab, Department of Physiotherapy, University of Thessaly, 3rd km Old National Road Lamia-Athens, 35132 Lamia, Greece; ggrammatikou@uth.gr (G.G.); nikstrimp@uth.gr (N.S.); 2 Department of Speech and Language Therapy, University of Ioannina, 4th km of Ioannina-Athens National Road, 45500 Ioannina, Greece; efterpipavlidou@uoi.gr

**Keywords:** preterm infants, early physiotherapy, parent-delivered intervention, family-centered care, motor development, neonatal intensive care unit, neurodevelopment, systematic review

## Abstract

**Background/Objectives:** Preterm birth is associated with increased risk of motor delay and broader neurodevelopmental vulnerability. Existing reviews have examined neonatal therapy, family-centered care, or early developmental intervention broadly, but the independent evidence for physiotherapy-based interventions in which parents actively deliver or support motor or sensorimotor activities remains uncertain. This review evaluated the effects of early physiotherapy with structured, guided parental involvement on motor development in preterm infants and examined intervention content, parental burden, safety, adherence, and durability of effects. **Methods:** The review followed PRISMA 2020 and a prespecified PICO framework. The original PubMed, Scopus, and PEDro search was updated through 9 July 2026 using PubMed/MEDLINE, PEDro, Cochrane CENTRAL, ClinicalTrials.gov, backward and forward citation tracking, and linked-report searches. Eligible studies were randomized controlled trials of early physiotherapy, motor, or structured sensorimotor intervention with an active parent-delivered or parent-supported component and a validated motor or neurodevelopmental outcome. Multiple publications from the same randomized cohort were linked and participants were counted once. Risk of bias was evaluated with Cochrane RoB 2, intervention reporting with TIDieR, and certainty with GRADE. **Results:** Ten reports represented five independent randomized cohorts and 393 unique randomized infants. The Norwegian Parent-Administered Physical Therapy Intervention (NOPPI) produced a moderate short-term improvement in TIMP performance at term-equivalent age, but no consistent advantage in general movements, motor performance at 3 or 24 months, or school-age motor outcomes. COPCA and a NICU-to-home physiotherapy program produced developmental improvement over time or selected short-term gains, without consistent superiority over active or developmental care comparators. A parent-administered sensorimotor intervention by Fucile et al. improved oral-feeding outcomes but not TIMP motor performance, and its linked 18-month follow-up did not demonstrate a clear developmental advantage. Badura et al. found no significant effect of a 10-week parent-delivered general-movement-based program on MOS-R or Bayley-III motor outcomes; adherence was variable, and a transient increase in maternal depressive symptoms at discharge highlighted the importance of treatment burden. **Conclusions:** Guided parental involvement is feasible and may enhance short-term motor performance when activities are individualized, active, cue-responsive, and closely supervised. However, current evidence does not establish sustained superiority over well-structured usual or traditional care, developmental normalization, or benefit from passive sensorimotor input alone. Clinical implementation should combine competency-based parent training, explicit infant stress cues and stopping rules, realistic dose targets, ongoing professional support, and monitoring of adherence, parental well-being, and adverse events.

## 1. Introduction

Preterm birth remains a major global child-health challenge. Approximately 13.4 million infants were born preterm in 2020, and survivors—particularly those born very or extremely preterm—remain at increased risk of motor, cognitive, behavioral, and participation-related difficulties [1,2,3,4,5]. Motor consequences range from subtle deficits in postural control, movement variability, reaching, balance, and locomotor acquisition to cerebral palsy and other persistent neuromotor disorders. Early motor behavior is clinically important because it supports exploration, perception–action learning, communication, and social participation rather than representing an isolated biomechanical domain.

The neonatal period and first post-term months are characterized by rapid synaptogenesis, myelination, cortical organization, and activity-dependent refinement. For infants born preterm, this sensitive period coincides with hospitalization, medical instability, constrained movement opportunities, and the formation of parent–infant interaction. Early intervention therefore needs to address not only which movement experiences are offered, but also their timing, frequency, variability, emotional context, and responsiveness to infant behavioral and physiologic cues [6,7,8,9].

Family-centered physiotherapy positions parents as partners rather than passive recipients of professional advice. Approaches such as COPCA emphasize infant autonomy, active exploration, caregiver coaching, and integration of therapeutic opportunities into ordinary routines [10]. Parent-administered programs may increase dose beyond scheduled clinical sessions, support contextual learning, and strengthen parental competence. Conversely, programs that are demanding, poorly individualized, or insufficiently responsive to infant cues may increase family burden and risk overly intrusive handling. Parent involvement should therefore be conceptualized as both a potential therapeutic mechanism and an implementation variable that requires training, supervision, and safety monitoring.

Previous evidence syntheses have addressed related but broader questions. Blauw-Hospers and Hadders-Algra reviewed the effects of early intervention on motor development more broadly [11]. Khurana et al. reviewed neonatal therapy across motor, cognitive, and behavioral domains [12]; Hughes et al. examined motor-development interventions in preterm infants [13]; Oh and Heo synthesized parenting interventions [14]; Raghupathy et al. evaluated family-centered care in very preterm infants [15]; and De Bruyn et al. focused on early interventions containing an active motor component [16]. Related parent-supported developmental approaches, including Supporting Play Exploration and Early Development Intervention (SPEEDI), have also been described in pilot, feasibility, and protocol publications [17,18,19]. Together, these bodies of evidence encompass heterogeneous professional disciplines, intervention targets, gestational-age groups, and levels of parental involvement. They do not fully answer the narrower clinical question of whether physiotherapy-based interventions in which parents are specifically trained or coached to deliver motor or sensorimotor opportunities improve validated motor outcomes in infants born at greatest developmental risk.

The present review therefore aimed to (1) determine the effects of early physiotherapy, motor, or structured sensorimotor interventions with guided parental involvement on motor development in preterm infants; (2) distinguish active infant practice from passive tactile, kinesthetic, or oral sensorimotor input; (3) examine whether reported gains indicate relative improvement or normative developmental catch-up; and (4) evaluate intervention dose, adherence, parental stress, training procedures, safety, and durability of parent-delivered practice.

## 2. Materials and Methods

### 2.1. Protocol and Reporting

This systematic review was designed and reported in accordance with the PRISMA 2020 statement [20]. The protocol was registered in PROSPERO (ID: 1137282). The protocol specified the population, intervention, comparators, outcomes, eligible designs, risk-of-bias assessment, and narrative synthesis. The updated review retained the original clinical question while explicitly linking multiple reports from the same randomized cohort to prevent double counting.

### 2.2. Eligibility Criteria

Eligibility criteria were structured using the PICO framework. The target population comprised infants born before 34 completed weeks of gestation or at 34 + 0 weeks when the trial explicitly classified the cohort as very/moderately preterm. Infants born at 34 + 1 to 36 + 6 weeks were not included unless separate data were available for the eligible subgroup. The threshold was selected because very and earlier-moderately preterm infants have greater neonatal exposure and motor vulnerability than the heterogeneous late-preterm population, and because combining these groups can dilute treatment effects and increase clinical heterogeneity. One contemporary trial used a combined entry criterion of gestational age <32 weeks or birth weight <1500 g [21]. It was retained because the cohort was described as very preterm and the reported gestational-age distribution was centered well below 32 weeks; the possibility that a small number of participants qualified by birth weight despite gestational age ≥34 weeks is addressed as a sensitivity limitation.

Eligible interventions were initiated during neonatal hospitalization or early infancy and included physiotherapy, motor intervention, or structured sensorimotor intervention in which parents or primary caregivers were trained, coached, supervised, or supported by a physiotherapist or rehabilitation professional to deliver or embed therapeutic activities. Interventions could contain active motor practice, positioning, handling, tactile or kinesthetic input, oral sensorimotor stimulation, or home-routine coaching, provided that a motor or neurodevelopmental outcome was measured. Comparators included usual neonatal care, developmental care, standard therapist-led physiotherapy, another active early intervention, or no additional intervention.

The primary outcome was motor development, including postural and selective motor performance, gross or fine-motor ability, spontaneous movement quality, or global motor development measured with validated instruments such as the Test of Infant Motor Performance (TIMP), General-Movement Assessment (GMA), Motor Optimality Score-Revised (MOS-R), Bayley Scales of Infant and Toddler Development, third edition (Bayley-III), Alberta Infant Motor Scale (AIMS), Peabody Developmental Motor Scales, second edition (PDMS-2), or Movement Assessment Battery for Children, second edition (MABC-2). Secondary outcomes included cognition, language, communication, personal–social development, parent–infant interaction, feeding, parental stress or depressive symptoms, adherence, fidelity, feasibility, and adverse events.

Randomized controlled trials published from 1 January 2014 through 9 July 2026 were eligible. The lower date limit was retained to focus on contemporary neonatal care and parent-partnership models; its implications for comprehensiveness were considered in the limitations. Reports were excluded when they lacked an active guided parental component, had no physiotherapy/motor/sensorimotor content, did not report a validated developmental outcome, included an inseparable ineligible population, or were conference abstracts without sufficient methodological detail.

### 2.3. Information Sources and Search Strategy

The original search used PubMed, Scopus, and PEDro and covered 2014–2024. A final update was conducted on 9 July 2026 using PubMed/MEDLINE, PEDro, Cochrane CENTRAL, ClinicalTrials.gov, backward reference checking, forward citation searching, and searches for trial registrations and linked publications. Search concepts included preterm or premature infants, very low birth weight, physiotherapy or physical therapy, early motor or sensorimotor intervention, parent- or caregiver-delivered intervention, family-centered care, motor development, neurodevelopment, and randomized trial terms. The complete copy-and-paste strategies for each source are provided in Supplementary Table S1; search material is deposited with the manuscript rather than being available only on request.

Search results were imported into Zotero and deduplicated. Title/abstract screening was followed by full-text assessment. Reference lists of included studies and recent systematic reviews were examined, and author names, registration identifiers, recruitment centers, dates, sample sizes, and intervention descriptions were cross-checked to identify companion reports from the same trial. The update deliberately searched from 1 January 2024 to capture articles published or indexed after the previous search cut-off.

### 2.4. Study Selection and Data-Extraction

Two reviewers independently screened records, assessed full texts, and extracted data using a standardized form. Disagreements were resolved by discussion and consensus; unresolved decisions were referred to a third reviewer. A formal inter-reviewer agreement statistic was not calculated. Extracted variables included study design, setting, participant characteristics, gestational age, sample size randomized and analyzed, intervention rationale, active or passive infant involvement, parent role, training procedures, dose, progression, adherence, fidelity, safety procedures, comparator content, outcomes, time points, attrition, and effect estimates. The full-text exclusion framework and primary reasons are provided in Supplementary Table S2, and the standardized data-extraction and appraisal fields are provided in Supplementary Table S3.

Reports were linked at the trial-cohort level using trial registration, recruitment sites and dates, investigators, sample size, baseline characteristics, intervention dose, and participant flow. When multiple reports analyzed the same randomized cohort, the trial was counted once in the number of independent studies and unique participants. Linked reports were retained because they contributed distinct outcomes or follow-up periods.

### 2.5. Risk of Bias and Certainty of Evidence

Risk of bias was assessed independently by two reviewers using the Cochrane Risk of Bias 2 (RoB 2) tool for individually randomized, parallel-group trials (version 22 August 2019; https://www.riskofbias.info/welcome/rob-2-0-tool/current-version-of-rob-2; accessed on 9 June 2026) [22]. Judgments covered the randomization process, deviations Corrected. The access date (9 July 2026) has been added for the official RoB 2 webpage.from intended interventions, missing outcome data, outcome measurement, and selection of the reported result. Disagreements were resolved by consensus and, when necessary, third-reviewer adjudication. Because intervention providers and parents could not be blinded, the assessment emphasized allocation procedures, blinded outcome assessment, attrition, analysis population, protocol availability, and selective reporting. Risk-of-bias judgments were assigned at the independent cohort/outcome level and summarized with a RoB 2 traffic-light figure. Detailed supporting judgments are provided in Supplementary Table S4. The RoB 2 traffic-light figure was generated using Matplotlib (version 3.10.8; Matplotlib Development Team; https://matplotlib.org/; accessed on 9 June 2026).

Certainty of evidence was evaluated using GRADE principles [23], considering risk of bias, inconsistency, indirectness, imprecision, and publication bias. Certainty was rated separately for short-term motor performance, spontaneous movement quality, longer-term standardized motor outcomes, broader development, and parent outcomes. Intervention descriptions were mapped to the TIDieR framework [24].

### 2.6. Data Synthesis

Meta-analysis was not undertaken because independent cohorts differed substantially in intervention content, theoretical orientation, infant participation, timing, dose, parental training, comparator intensity, outcome instruments, scoring metrics, and follow-up. Findings were synthesized narratively by independent cohort and outcome domain. Direct trial findings were distinguished from mechanistic interpretation and clinical inference. Particular attention was given to report overlap, active versus passive intervention content, normative interpretation, adherence, maintenance after formal support ended, parental stress, and safety. The completed PRISMA 2020 checklist and location map are provided in Supplementary Table S5.

## 3. Results

### 3.1. Study Selection and Cohort Linkage

**Figure 1:**
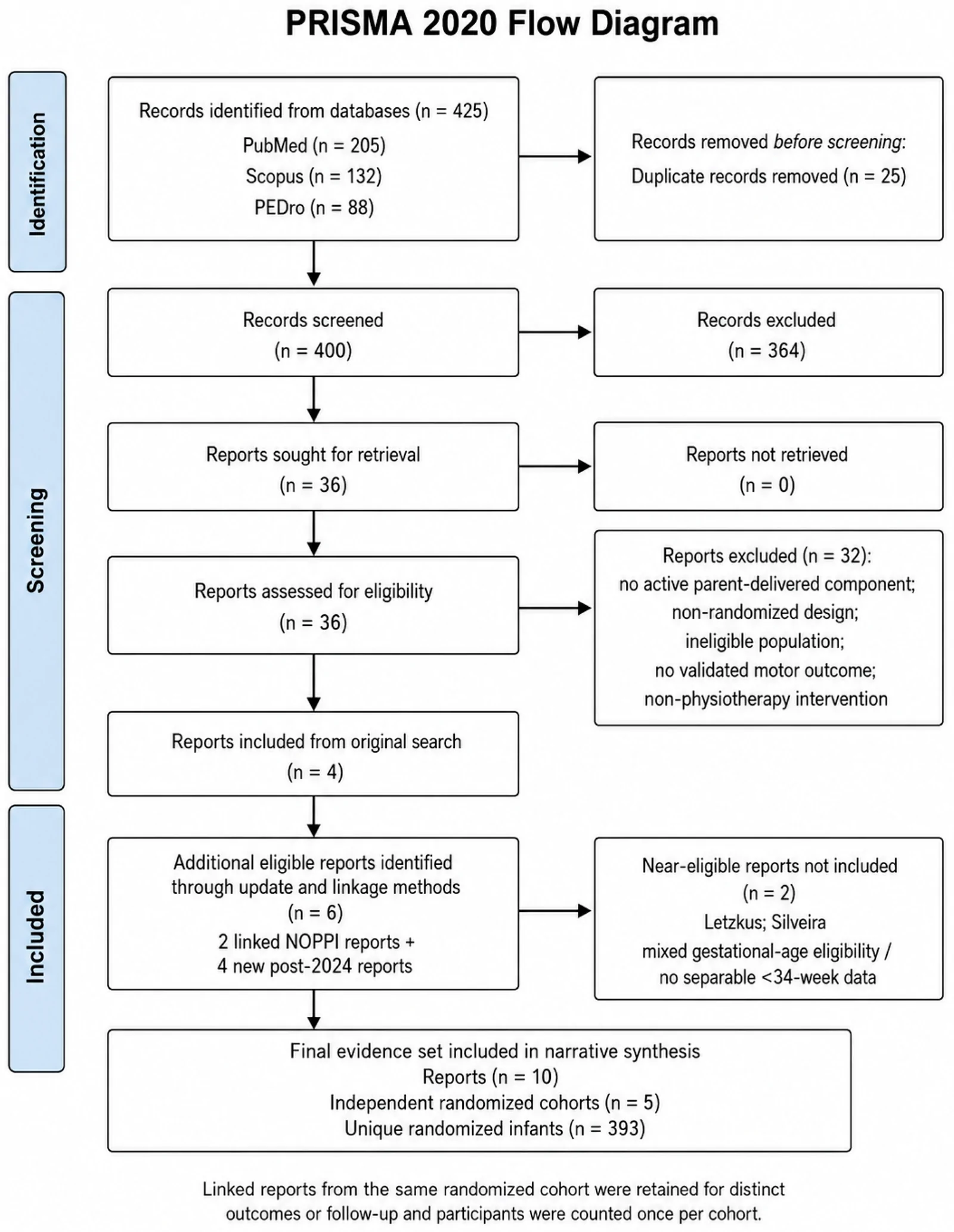
PRISMA 2020 study selection and report-linkage flow. The diagram distinguishes reports from independent randomized cohorts and prevents double counting of participants in linked publications.

The original database search identified 425 records (PubMed, n = 205; Scopus, n = 132; PEDro, n = 88). After removal of 25 duplicates, 400 records were screened, 36 full-text reports were assessed, and four reports met the original eligibility criteria. The updated search and linked-publication assessment added two NOPPI outcome reports that had previously been cited only as contextual evidence and four reports published after the former search cut-off. The final evidence set comprised 10 reports from five independent randomized cohorts involving 393 unique randomized infants. Participants in linked NOPPI and PASI publications were counted once. Two near-eligible trials (Letzkus and Silveira) were not included because the mixed gestational-age/birth-weight eligibility criteria did not allow confirmation of a fully eligible <34-week population or separable subgroup data. The updated report-level selection and linkage process is shown in Figure 1.

### 3.2. Characteristics of Included Cohorts and Reports

Five independent randomized cohorts were conducted in Norway, Türkiye, Spain, Canada, and Germany. Interventions ranged from a 10-day predominantly passive tactile/oral sensorimotor program to 10 weeks of parent-delivered general-movement-based therapy, and from a brief NICU program to a 9-month family-coaching model. Comparator intensity varied from usual neonatal care to traditional early intervention physiotherapy and NIDCAP-based developmental care. Table 1 summarizes cohort-level characteristics and identifies linked reports.

### 3.3. Intervention Content, Infant Activity, and Parental Training

The interventions should not be treated as equivalent forms of infant massage. NOPPI and COPCA primarily sought active infant participation. Parents adjusted support, waited for responses, facilitated postural control, and created opportunities for self-initiated movement. Ochandorena-Acha et al. combined passive tactile and joint-movement input during NICU hospitalization with more active positioning and movement opportunities at home. PASI was predominantly passive and regulation-focused, consisting of stroking the limbs and trunk, oral stimulation, and non-nutritive sucking. The Badura intervention involved externally guided imitation of general-movement patterns; although movement was generated through parent handling, the protocol aimed to provide variable sensorimotor feedback rather than infant-led problem-solving.

Parent preparation ranged from direct demonstration and supervised practice to coaching, prerecorded education, written or video materials, and telehealth follow-up. Competency testing and fidelity monitoring were inconsistent. NOPPI documented delivered dose, and Badura et al. used parent diaries and a feasibility questionnaire; only 63.6% of participating families achieved medium or high adherence in the latter trial. PASI incorporated physiologic and behavioral stopping rules and reported no adverse events in parent logs. Table 2 provides an abbreviated TIDieR-based comparison.

### 3.4. Risk of Bias

**Figure 2:**
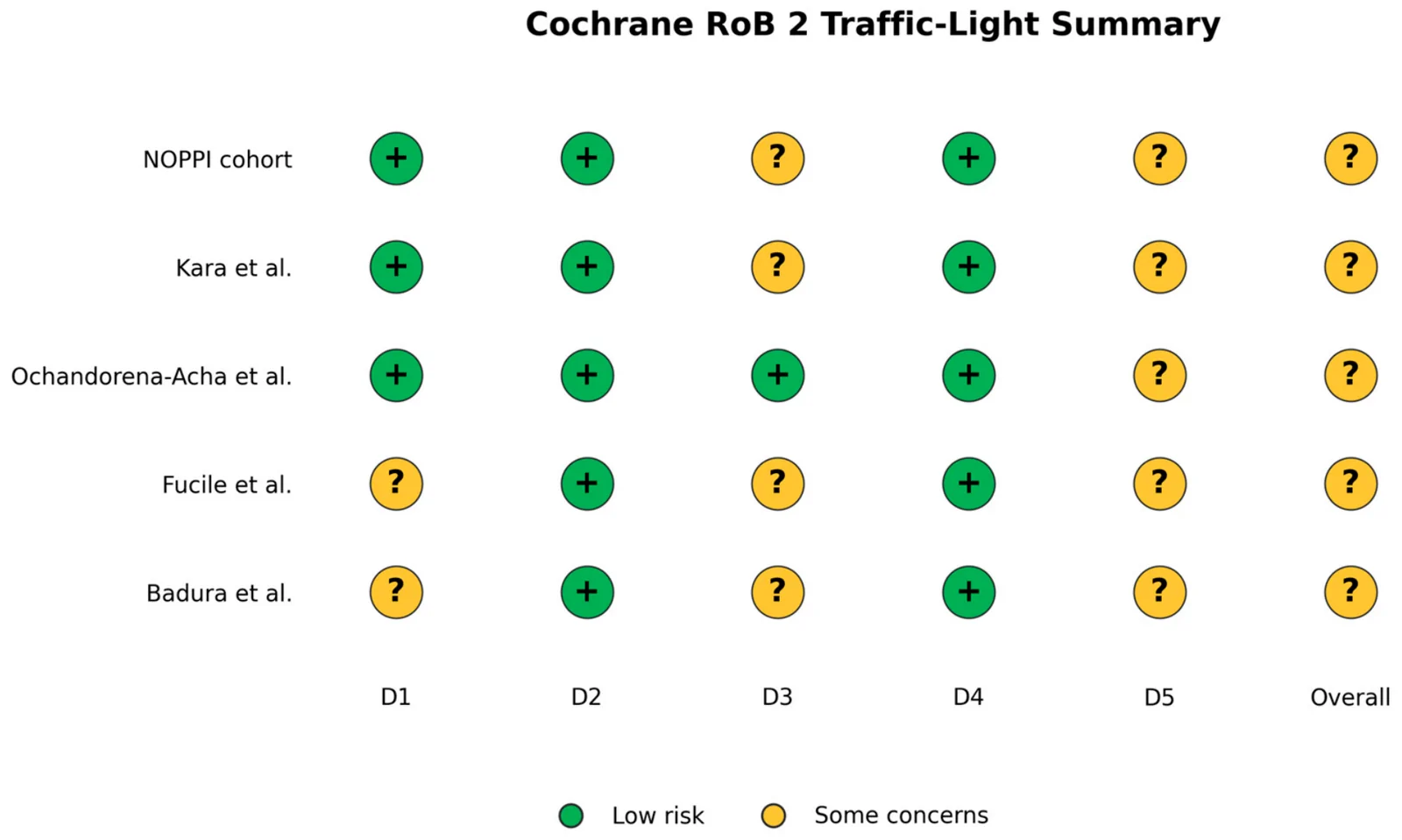
Cochrane RoB 2 traffic-light summary [22]. D1, randomization process; D2, deviations from intended interventions; D3, missing outcome data; D4, outcome measurement; D5, selection of the reported result. “+” denotes low risk, and “?” denotes some concerns.

No independent cohort was judged to have high overall risk of bias. All cohorts raised at least some concerns, most commonly because of incomplete availability of prespecified statistical analysis plans, attrition at later follow-up, or uncertainty about selective outcome reporting. Randomization was generally adequate, although the Fucile report provided limited public detail on sequence concealment and the Badura trial assigned twins to the same group after use of a randomization list, creating some concern about the randomization process. Blinded motor assessment was reported in several studies, including MOS-R and Bayley-III assessment in the Badura trial. The inability to blind parents and therapists was inherent to the intervention and was not, by itself, considered sufficient for high risk. Figure 2 summarizes the RoB 2 judgments; detailed rationales are provided in Supplementary Table S4.

### 3.5. Effects on Motor and Developmental Outcomes

#### 3.5.1. Short-Term Motor Performance and Spontaneous Movement Quality

In the NOPPI cohort, Ustad et al. reported a statistically significant improvement in TIMP performance at 37 weeks PMA after the three-week parent-administered program. The adjusted between-group difference in TIMP z score was 0.42 (95% CI 0.13 to 0.72; *p* = 0.005), corresponding to a moderate standardized effect (d approximately 0.40) [25]. This was the clearest positive motor finding in the evidence base. However, Fjørtoft et al. did not identify significant between-group differences in fidgety movements or movement character at three months of corrected age in a subset of the same randomized cohort [26]. The 2020 linked report similarly found no group difference in TIMP z score at three months, although greater delivered intervention dose was positively associated with TIMP performance [27].

Fucile et al. randomized 94 infants to PASI or standard care. PASI improved feeding outcomes, including earlier attainment of complete oral feeding and a greater frequency of direct breastfeeding at discharge, but TIMP motor scores were virtually identical between groups (46.9 [SD 4.8] vs 46.8 [SD 8.4]; *p* = 0.961) [32]. This finding is important because it separates benefit for the intervention’s primary oral-feeding target from a generalized motor effect. The predominantly passive tactile/oral program should not be interpreted as evidence that infant massage or passive stimulation improves gross motor development.

Badura et al. found no significant difference in MOS-R at three-to-five months of corrected age. Median MOS-R was 24 in both groups, and the adjusted intervention coefficient did not indicate benefit [21]. The trial reported that three infants with poor neuromotor features before treatment had favorable later outcomes, but all were in the intervention group and the subgroup was too small for causal inference. Overall, spontaneous movement evidence did not demonstrate a consistent effect of parent-delivered intervention.

#### 3.5.2. Motor Outcomes Beyond Early Infancy

Kara et al. observed improvement in Bayley-III fine and gross motor scores over time in both COPCA and traditional early intervention groups, but COPCA was not statistically superior to the active comparator [30]. Ochandorena-Acha et al. reported early improvement in selected ASQ-3 fine-motor and broader domains; AIMS scores did not demonstrate a consistent between-group advantage at later follow-up [31].

In the NOPPI cohort, no significant intervention–control difference was found on PDMS-2 at 24 months of corrected age. Nevertheless, higher intervention dose was associated with better Gross Motor and Total Motor scores, and both groups showed declining relative motor performance between 6 and 24 months [28]. At 7–10 years, 92 children from the original NOPPI cohort (43 intervention, 49 control) were assessed, and motor outcomes again did not differ between randomized groups [29]. These linked reports indicate that the short-term TIMP effect did not translate into demonstrated long-term superiority.

The linked PASI follow-up evaluated a subset of the original cohort at 4 and 18 months. Direct breastfeeding remained more frequent at 4 months, but no clear group difference in standardized developmental outcomes was demonstrated at 18 months; parents in the control group reported more developmental concerns, a finding that is hypothesis-generating and vulnerable to respondent and attrition bias [33]. Badura et al. found no significant group differences in Bayley-III motor, cognitive, or language outcomes at 18–24 months. Median motor composite scores were similar in the intervention and control groups [21].

#### 3.5.3. Normative Development and Developmental Catch-Up

The included trials primarily tested relative differences between randomized groups and did not establish sustained normalization to term-born developmental expectations. A statistically significant TIMP z-score difference in NOPPI indicates relative short-term improvement, but the later absence of group differences and the decline in relative motor performance from 6 to 24 months argue against assuming durable catch-up [25,27,28,29]. Fucile et al. reported equivalent TIMP scores rather than improved normative status [32]. Bayley-III and AIMS improvement over time in Kara and Ochandorena-Acha may reflect maturation and exposure to active care in both arms; neither trial demonstrated that infants reached or maintained term-born norms [30,31].

Badura et al. reported Bayley-III central values near the normative mean, but cohort-level averages cannot establish normalization for individual infants, and there was no randomized treatment advantage [21]. Future trials should report standardized scores, proportions below clinically meaningful cut-offs, reliable change, and comparison with appropriate normative or term-born reference groups rather than relying solely on within-group maturation.

#### 3.5.4. Parental Stress, Training, Adherence, and Safety

Parental outcomes were inconsistently measured. Ochandorena-Acha et al. included the Parenting Stress Index-Short Form, but the available evidence did not demonstrate a robust or sustained intervention effect on parental stress [31]. Badura et al. provided the most detailed contemporary assessment. The intervention did not improve parental mental health overall, and maternal depressive symptoms were transiently worse at hospital discharge before converging by three-to-five months of corrected age [21]. Qualitative feedback indicated that parents generally felt supported and empowered, yet several found the required frequency difficult to maintain. This tension shows that training opportunities can reduce uncertainty while an unrealistic daily dose can increase burden.

No included trial demonstrated that stressed parents harmed an infant through intrusive handling. However, samples were too small to estimate rare adverse events, and adverse-event reporting was not equally detailed. PASI specified that the session should be stopped for tachycardia, bradycardia, tachypnea, desaturation, stiffening, grimacing, yawning, sneezing, crying, hiccuping, gaze aversion, or eye closure, and no major adverse events were reported in parent logs [32]. Such cue-based stopping rules, supervised practice, and explicit contraindications provide a stronger safety model than unsupervised exercise prescription.

The duration for which parents continued learned activities after formal support ended was rarely measured. NOPPI dose–response findings and the low adherence in Badura et al. suggest that delivered exposure may be more informative than assigned group. The absence of maintenance data limits interpretation of fading effects and should be considered a central outcome in future trials. The developmental, normative, and family-level findings are summarized in Table 3.

### 3.6. Certainty of Evidence

Certainty was low for short-term motor performance and spontaneous movement outcomes and low-to-very-low for longer-term motor, broader developmental, parental, and safety outcomes. Downgrading reflected risk-of-bias concerns, small samples, attrition, clinical and methodological heterogeneity, inconsistent direction of findings, and imprecision. Publication bias could not be excluded because the number of independent cohorts was too small for formal assessment and gray-literature searching remained limited. Table 4 presents the GRADE summary.

## 4. Discussion

### 4.1. Principal Findings

This review identified 10 reports from five independent randomized cohorts involving 393 unique infants. Linking reports to their source cohort materially changes interpretation: the Ustad, Fjørtoft, Øberg, and Paulsen publications describe different outcomes and follow-up periods from the same NOPPI trial rather than multiple independent trials. The evidence base is therefore broader in follow-up duration than in independent replication.

The most convincing positive motor result was the short-term NOPPI improvement in TIMP at term-equivalent age. That effect was not accompanied by later randomized-group differences in general movements, TIMP at three months, PDMS-2 at two years, or school-age motor function. Fucile et al. showed that a parent-administered sensorimotor program can improve oral feeding while leaving TIMP unchanged, illustrating the importance of matching conclusions to the intervention target. Badura et al. extended parent delivery into the home with telehealth support but found no overall MOS-R or Bayley-III benefit and documented practical limits in adherence. Across studies, parent-guided physiotherapy was feasible and acceptable to many families, but superiority over good, usual, or active care was not consistently demonstrated.

### 4.2. Active Versus Passive Intervention Content

The distinction between active and passive content helps interpret heterogeneous findings. NOPPI and COPCA were organized around active, response-contingent motor behavior: the infant was expected to orient, stabilize, move, or solve a postural problem within graded support. PASI delivered valuable tactile and oral sensorimotor input but was not designed primarily as active gross motor practice; its feeding benefit and null TIMP results are therefore coherent rather than contradictory. Ochandorena-Acha combined passive and active components, while the Badura protocol used externally guided general-movement imitation with variable opportunities for infant contribution.

The direct evidence supports intervention packages, not isolated ingredients. It is biologically plausible that active, variable, task-relevant, and cue-responsive practice is more likely to influence motor learning than passive handling alone, but the current trials do not permit a component-level causal conclusion. Future factorial or process-evaluation designs should quantify how much time infants spend actively generating movement, how parents grade assistance, and whether change in active practice mediates outcome.

### 4.3. Dose, Adherence, and Maintenance

Assigned dose and delivered dose were not equivalent. NOPPI prescribed two sessions daily and identified positive dose–response associations at three and 24 months despite null randomized-group differences at those time points. Badura et al. prescribed three daily sessions for 10 weeks, but only 63.6% of families achieved medium or high adherence and the median number of sessions was substantially below the theoretical maximum. These findings indicate that demanding schedules may reduce implementation sufficiently to dilute efficacy.

The studies rarely examined how long parents continued using learned strategies after formal intervention or follow-up ended. This omission is clinically important because a short NICU program may alter immediate performance without changing the cumulative developmental environment. Maintenance should be measured using logs, app-supported tracking, periodic observation, and interviews that distinguish intentional therapeutic practice from naturally occurring caregiving. Future analyses should report both intention-to-treat effects and exposure-response relationships without treating non-randomized dose associations as causal.

### 4.4. Normative Development and Clinical Meaning

A gain relative to a comparator is not synonymous with catching up to term-born norms. NOPPI improved short-term standardized TIMP performance, but longer follow-up did not demonstrate sustained group separation. Improvement over repeated Bayley-III or AIMS assessments can reflect maturation, regression toward the mean, or effective care in both groups. The field should report the proportion of infants moving above clinically relevant thresholds, reliable change indices, and participation-level outcomes in addition to mean scores.

Outcome selection also influences conclusions. TIMP is sensitive to early postural and selective motor control; GMA and MOS-R characterize spontaneous movement quality and early neurological risk; AIMS describes gross motor maturation; Bayley-III and PDMS-2 measure broader standardized abilities; MABC-2 assesses later functional motor competence. These measures are complementary rather than interchangeable [34,35]. A core outcome set spanning early motor performance, later functional motor ability, parent-infant interaction, participation, and family burden would improve comparability.

### 4.5. Family Stress, Training Opportunities, and Safety

Parent involvement can reduce helplessness by providing knowledge and a meaningful role, but it can also shift responsibility onto families already coping with uncertainty, feeding difficulties, appointments, fatigue, and socioeconomic constraints. Related dyadic programs have reported benefits for parental symptoms [36,37,38], whereas the physiotherapy trials reviewed here did not show a consistent stress reduction. Badura et al. observed a transient worsening in maternal depressive symptoms at discharge, plausibly related to study demands and the prescribed frequency [21]. This finding argues against equating more sessions with better family-centered care.

Training should be a longitudinal clinical process rather than a single instruction session. At minimum, it should include demonstration, supervised practice, teach-back, competency assessment, written or video resources, repeated opportunities for questions, and follow-up after discharge. Parents should learn physiologic and behavioral stress cues, safe handling boundaries, explicit stopping rules, and when to contact the clinical team. The Fucile protocol offers a useful example of cue-based discontinuation criteria [32].

The theoretical risk that a highly stressed parent could use excessively intrusive physical handling cannot be dismissed simply because no injury was reported. Existing trials were not powered to detect rare harms and adverse-event definitions were inconsistent. Risk mitigation requires individualized contraindication screening, observation of parent technique, realistic dosing, rapid access to professional advice, and systematic reporting of desaturation, cardiorespiratory instability, sustained distress, musculoskeletal injury, feeding disruption, and caregiver psychological burden.

### 4.6. Clinical Implications

Clinical recommendations should remain proportional to the evidence. The NOPPI cohort supports the feasibility and short-term efficacy of individualized, parent-administered active motor practice during NICU hospitalization [25], but later linked reports show that sustained superiority cannot be assumed [26,27,28,29]. COPCA can be implemented as autonomy-oriented coaching, yet it was not superior to traditional early intervention [30]. The Ochandorena-Acha program supports a structured NICU-to-home pathway but produced limited later motor separation [31]. PASI supports parent delivery for oral-feeding goals, not for generalized motor improvement [32,33]. Badura et al. demonstrate that telehealth-supported home continuation is feasible, while also showing that adherence and parental burden can constrain effect [21].

Accordingly, neonatal and pediatric physiotherapists may reasonably involve parents early, embed brief opportunities in daily routines, and prioritize active, individualized, cue-responsive participation. These are evidence-informed implementation principles rather than proof that any single component is superior. The physiotherapist remains responsible for assessment, contraindication screening, parent competency, progression, fidelity, safety, and adjustment of the program to infant state and family capacity. Passive massage or repetitive handling should not be presented as an established motor-development treatment when the evidence supports a different target or remains null.

### 4.7. Strengths and Limitations

The main strength of this review is its focused clinical question and explicit separation of reports from independent randomized cohorts. The synthesis distinguishes active from passive infant involvement, links conclusions to specific trials, evaluates normative interpretation, and incorporates structured TIDieR, RoB 2, and GRADE summaries. The updated search added contemporary evidence on passive sensorimotor input, telehealth-supported general-movement therapy, long-term NOPPI outcomes, adherence, and parental mental health.

Several limitations remain. First, only five independent randomized cohorts and 393 unique infants were identified, and several outcomes relied on one cohort. Second, interventions, comparators, instruments, and follow-up periods were too heterogeneous for meaningful meta-analysis. Third, the 2014 lower date limit may have omitted older eligible trials, although it was chosen to reflect contemporary neonatal care. Fourth, CINAHL, Embase, and Web of Science were not available in the update, and gray literature was not comprehensively searched; publication bias and missed studies remain possible. Fifth, the Badura trial used eligibility based on gestational age <32 weeks or birth weight <1500 g. Although the reported cohort was centered well below 32 weeks, the absence of a maximum gestational age means that complete conformity with the <34-week threshold cannot be guaranteed. Sixth, adherence, fidelity, maintenance, parental burden, and adverse events were incompletely reported, and linked follow-up reports were vulnerable to attrition. Finally, the evidence does not permit confident component-level conclusions or subgroup effects by brain injury, gestational age, baseline movement abnormality, or family stress.

### 4.8. Future Research

Future trials should be prospectively registered, adequately powered, and reported according to CONSORT and TIDieR. Eligibility should define gestational age and birth-weight criteria transparently and report separable subgroup data. Interventions should quantify active infant-generated movement, passive input, dose progression, parent competency, fidelity, adherence, maintenance, and co-interventions. Usual care should be described in sufficient detail to interpret between-group effects.

Longer follow-up is essential but should not be limited to repeated impairment-level testing. Outcomes should include functional motor ability, cognition, language, participation, parent-infant interaction, parental stress and self-efficacy, quality of life, and adverse events. Trials should predefine minimal clinically important differences and normative thresholds. Adaptive designs may be particularly useful for targeting infants with abnormal early movement patterns or brain injury while reducing unnecessary burden for infants with low baseline risk. Digital support may improve continuity, but its value should be tested against caregiver workload and equity of access.

## 5. Conclusions

Ten reports from five independent randomized cohorts and 393 unique infants indicate that guided parental involvement in early physiotherapy is feasible and may improve short-term motor performance in selected contexts. The clearest benefit was a moderate TIMP improvement after a brief individualized NICU program; this did not translate into consistent later superiority. A predominantly passive parent-administered sensorimotor program improved feeding but not motor performance, and a 10-week telehealth-supported general-movement program did not improve MOS-R or Bayley-III outcomes. Current evidence therefore supports cautious, family-sensitive implementation rather than a conclusion that parent-guided physiotherapy is universally superior to well-structured care. Programs should emphasize individualized active practice when motor learning is the target, competency-based parent education, explicit safety and stopping rules, realistic dose expectations, and monitoring of adherence and parental well-being.

## Figures and Tables

**Table 1:** Characteristics of independent randomized cohorts and linked outcome reports.

Cohort/Linked Reports	Randomized Sample and Gestational Age	Intervention and Parent Role	Dose/Timing	Comparator	Outcomes and Follow-Up	Main Trial-Level Finding
NOPPI, Norway: Ustad 2016 [25]; Fjørtoft 2017 [26]; Øberg 2020 [27]; Øberg 2022 [28]; Paulsen 2026 [29]	153 infants; GA ≤32 weeks. Follow-up samples: 130 at 3 months for GMA; 92 at 7–10 years.	Parents delivered individualized active motor activities after physiotherapist instruction and supervision; head/trunk control, midline orientation, antigravity and postural responses.	3 weeks at 34–36 weeks PMA; 2 × 10 min/day.	Usual NICU care.	TIMP at term and 3 months; GMA at 3 months; PDMS-2 at 24 months; MABC-2/long-term motor assessment at 7–10 years.	Short-term TIMP benefit at term-equivalent age; no consistent between-group benefit at later time points. Higher delivered dose was associated with better motor scores in secondary analyses.
Kara et al., Türkiye [30]	32 infants (16/16); GA ≤32 weeks with abnormal GMA.	COPCA coaching emphasized infant autonomy, variability, problem-solving, active exploration, and parent use of daily routines.	2 sessions/week, 1 h/session, for 9 months.	Traditional early intervention physiotherapy.	Bayley-III fine and gross motor scales at 3, 6, 9, 12, and 24 months corrected age.	Both groups improved; COPCA was not statistically superior to traditional physiotherapy.
Ochandorena-Acha et al., Spain [31]	48 infants (24/24); GA 28 + 0 to 34 + 0 weeks.	Physiotherapy plus parent education, handling guidance, developmental information, and home activities; passive tactile/kinesthetic NICU input plus active home opportunities.	From 32 weeks PMA to 2 months corrected age; NICU and home.	Routine/NIDCAP-based developmental care.	AIMS, ASQ-3, and PSI-SF at 1, 2, and 8 months corrected age.	Selected early ASQ-3 gains; no consistent later between-group AIMS advantage.
PASI, Canada: Fucile et al. [32]; Smith et al. [33]	94 randomized; mean GA 31.0 weeks (SD 2.2); 80 completed the neonatal study.	Parents provided tactile input to trunk/limbs, oral-structure stimulation, and non-nutritive sucking. Infant participation was predominantly passive/regulated rather than active motor problem-solving.	15 min once daily for 10 days, 15–30 min before feeding.	Standard NICU care.	Feeding milestones and TIMP in NICU; direct breastfeeding and developmental/parent-report outcomes at 4 and 18 months.	Earlier full oral feeding and more direct breastfeeding; no neonatal TIMP difference and no clear 18-month developmental advantage.
GeMo-Support, Germany: Badura et al. [21]	66 infants (32 control/34 intervention); eligibility GA <32 weeks or BW <1500 g; cohort median GA 29.0 weeks (IQR 26.3–30.0).	Parents were trained by pediatric physiotherapists to imitate age-specific general-movement patterns; prerecorded education and weekly telehealth support after discharge.	3 times/day for 10 weeks, starting at 34 weeks PMA.	Usual care including physiotherapy 1–3 times/week as clinically indicated.	MOS-R at 3–5 months; Bayley-III at 18–24 months; EPDS/WEMWBS; adherence and feasibility.	No significant neurodevelopmental advantage. Adherence was variable; transiently worse maternal depressive symptoms were observed at discharge but not later.

**Table 2:** Abbreviated TIDieR-based comparison of intervention components.

TIDieR Domain	NOPPI	COPCA	Ochandorena-Acha Program	PASI (Fucile)	GM-Based Program (Badura)
Rationale	Improve postural control and motor performance through repeated individualized practice.	Promote variability, autonomy, problem-solving, and family participation.	Support motor/global development and parent competence across NICU-to-home transition.	Enhance oral feeding through tactile, oral, and sucking-related sensorimotor input; motor function was also assessed.	Increase variable sensory feedback by imitating age-specific general-movement patterns and strengthen parent self-efficacy.
Infant activity	Predominantly active, response-contingent postural and transitional activity.	Active, infant-initiated exploration embedded in routines.	Mixed passive NICU input and active home motor opportunities.	Predominantly passive tactile/oral stimulation and non-nutritive sucking.	Parent-guided limb/trunk movement; active infant contribution was variable and not the principal mechanism.
Parent role	Deliver individualized activities twice daily after physiotherapist training.	Use coaching principles and adapt routines rather than reproduce fixed exercises.	Provide handling, positioning, play, and developmental activities.	Administer a standardized 15 min sequence before feeding.	Administer therapy three times daily and continue after discharge with telehealth support.
Training/support	Instruction, demonstration, supervision, and repeated contact with physiotherapists.	Ongoing coaching during family-centered sessions.	Education, demonstration, written guidance, and home follow-up.	Instruction and practice; explicit observation of infant stability and stopping rules.	Pediatric physiotherapist training, prerecorded video, and weekly video conferences after discharge.
Dose	2 × 10 min/day for 3 weeks.	2 × 60 min/week for 9 months.	From 32 weeks PMA to 2 months corrected age; variable NICU/home exposure.	15 min/day for 10 days.	3 sessions/day for 10 weeks.
Adherence/fidelity	Dose recorded; higher dose associated with better later scores, but maintenance after discharge was uncertain.	Process-oriented; adherence and fidelity not uniformly quantified.	Not consistently quantified across phases.	Most sessions delivered by mothers; parent logs reported no major adverse events.	Median 64 sessions over 10 weeks; only 63.6% achieved medium/high adherence; several daily sessions were burdensome.
Safety	Professional supervision and individualized progression.	Cue-responsive coaching and infant autonomy.	Handling guidance; adverse-event reporting limited.	Stop for physiologic or behavioral stress signs; no adverse events reported.	Feasible and well tolerated; parental burden and mental health were monitored.

**Table 3:** Summary of developmental and family outcomes by independent cohort.

Cohort/Outcome	Main Result	Normative Interpretation	Parent/Implementation Finding	Certainty
NOPPI: TIMP at term [25]	Adjusted TIMP z-score difference 0.42 (95% CI 0.13–0.72), *p* = 0.005; moderate short-term effect.	Relative short-term improvement; sustained normalization not established.	Parents delivered 2 × 10 min/day under physiotherapist guidance.	Low
NOPPI: GMA/TIMP at 3 months [26,27]	No randomized-group difference; higher delivered dose associated with better TIMP scores.	No evidence of normalized spontaneous movement quality.	Dose varied; assigned group alone did not explain later outcome.	Low
NOPPI: PDMS-2 and school-age motor [28,29]	No group difference at 24 months or 7–10 years; dose association at 24 months.	Long-term catch-up attributable to intervention not demonstrated.	Post-program maintenance was not adequately measured.	Low
Kara: Bayley-III [30]	Both groups improved; COPCA not superior to traditional physiotherapy.	Improvement over time did not demonstrate normalization.	Coaching supported active routines; fidelity/adherence incompletely quantified.	Very low
Ochandorena-Acha: AIMS/ASQ-3/PSI-SF [31]	Selected early ASQ-3 gains; no consistent later AIMS advantage.	Short-term screening gains did not establish sustained catch-up.	Mixed NICU/home program; parent-stress benefit uncertain.	Very low
PASI: TIMP and 18-month development [32,33]	No TIMP difference; no clear standardized developmental benefit at 18 months.	No motor normalization effect demonstrated.	Feeding improved; explicit stress-cue stopping rules; parent-reported concerns differed at follow-up.	Low for TIMP; very low for follow-up
Badura: MOS-R/Bayley-III [21]	No significant motor or broader neurodevelopmental difference.	Scores near normative mean in some domains, but no treatment-attributable normalization.	Variable adherence; transiently worse maternal depressive symptoms at discharge.	Low

**Table 4:** GRADE summary of findings and reasons for downgrading.

Outcome Domain	Independent Cohorts/Participants	Risk of Bias	Inconsistency	Indirectness	Imprecision/Publication Bias	Overall Certainty
Short-term motor performance (TIMP)	2 cohorts; NOPPI n = 153, PASI n = 94	Serious: reporting/attrition concerns	Serious: one positive and one null trial with different intervention targets	Serious: PASI primarily targeted feeding	Serious: small evidence base; publication bias cannot be assessed	Low
Spontaneous movement quality (GMA/MOS-R)	2 cohorts; NOPPI follow-up n = 130, Badura n = 66	Serious	Not serious: both broadly null	Serious: different constructs and timing	Serious: small samples and few abnormal cases	Low
Motor development at 18–24 months	4 cohorts with variable follow-up	Serious: attrition and selective-reporting concerns	Serious: heterogeneous measures and comparators	Not serious to serious	Serious: small cohorts and wide uncertainty	Low
School-age motor outcome	1 cohort; n = 92 follow-up	Serious: attrition from original cohort	Not estimable	Not serious	Very serious: single cohort	Very low
Broader developmental outcomes	3 cohorts	Serious	Serious	Serious: often secondary or parent-reported	Serious	Very low
Parental stress/mental health	2 cohorts	Serious	Serious	Not serious	Very serious: small samples and inconsistent time points	Very low
Safety and adverse events	5 cohorts, inconsistently reported	Serious	Not estimable	Serious: rare harms not targeted	Very serious; probable reporting bias	Very low

## Data Availability

No new participant-level dataset was created. Review materials and Supplementary Files accompany the submission; study data are available in the original reports.

## Supplementary Materials

The following supporting information can be downloaded at https://www.mdpi.com/article/10.3390/pediatric18040100/s1, Table S1: complete database and registry search strategies; Table S2: full-text exclusion framework and primary reasons; Table S3: standardized data-extraction and appraisal form; Table S4: detailed Cochrane RoB 2 supporting judgments; and Supplementary Table S5, PRISMA 2020 checklist and location map.
