# Supplementary material for: Effectiveness of Early Physiotherapy Interventions with Guided Parental Involvement on Motor Development in Preterm Infants: A Systematic Review
Source: Pediatr Rep. 2026 Jul 29;18(4):100. doi: 10.3390/pediatric18040100 (PMC13516984; doi:10.3390/pediatric18040100)
Supplement: Supplementary file 1 [file pediatrrep-18-00100-s001.zip › pediatrrep-4406108-supplementary.pdf]

SUPPLEMENTARY MATERIALS

Effectiveness of Early Physiotherapy Interventions with Guided Parental Involvement on Motor Development in Preterm Infants: A Systematic Review

Georgios Grammatikou, Efterpi Pavlidou, Nikolaos Strimpakos, and Konstantinos Chandolias

Final corrected submission to Pediatric Reports | Manuscript ID: pediatrrep-4406108

Contents

• Supplementary Table S1. Complete database and registry search strategies
• Supplementary Table S2. Full-text exclusion framework and primary reasons
• Supplementary Table S3. Standardized data-extraction and appraisal form
• Supplementary Table S4. Detailed Cochrane RoB 2 supporting judgments
• Supplementary Table S5. PRISMA 2020 checklist and location map
• Supplementary References

Final literature-search date: 9 July 2026

This file contains five supplementary tables (Tables S1–S5). Reference numbering in this supplementary file follows the citation numbers used in the revised main manuscript.

Supplementary Table S1. Complete Database and Registry Search Strategies

The original search covered PubMed, Scopus, and PEDro for publications dated 1 January 2014 to 31 December 2024. During revision, the search was updated through 9 July 2026 and expanded to Cochrane CENTRAL and ClinicalTrials.gov. Searches were conducted without language restrictions. Reference-list screening, forward citation searching, and trial-registration checks were also undertaken. The original audited database counts were PubMed (*n* = 205), Scopus (*n* = 132), and PEDro (*n* = 88). The update identified one additional eligible randomized report; the update arm is presented separately in the PRISMA flow diagram so that the original audited counts remain transparent.

Source	Coverage	Last searched	Limits / notes
PubMed (MEDLINE)	1 Jan 2014–9 Jul 2026	9 Jul 2026	No language limit; human studies; randomized/controlled-study concepts
Scopus	1 Jan 2014–9 Jul 2026	9 Jul 2026	Title/abstract/keyword search; publication years 2014–2026
PEDro	2014–2026	9 Jul 2026	Advanced Search; paediatrics; clinical trial
Cochrane CENTRAL	2014–2026	9 Jul 2026	Trials search; title/abstract/keyword fields
ClinicalTrials.gov	All records posted/updated through 9 Jul 2026	9 Jul 2026	Interventional studies; child age group; all recruitment statuses
Other sources	Reference lists and forward citations	9 Jul 2026	Included reports, key reviews, linked NOPPI reports, and registrations

PubMed

((("Infant, Premature"[Mesh] OR preterm infant*[tiab] OR premature infant*[tiab] OR very preterm[tiab] OR extremely preterm[tiab] OR very low birth weight infant*[tiab]) AND ("Physical Therapy Modalities"[Mesh] OR physiotherap*[tiab] OR physical therap*[tiab] OR motor intervention*[tiab] OR motor training[tiab] OR sensorimotor intervention*[tiab] OR developmental intervention*[tiab] OR early intervention*[tiab]) AND (parent*[tiab] OR caregiver*[tiab] OR family-centered[tiab] OR family-centred[tiab] OR parent-delivered[tiab] OR parent-administered[tiab] OR parent-guided[tiab] OR home program*[tiab] OR home programme*[tiab] OR coaching[tiab]) AND (motor development[tiab] OR neurodevelopment*[tiab] OR TIMP[tiab] OR general movement*[tiab] OR Bayley[tiab] OR AIMS[tiab] OR MOS-R[tiab]) AND (randomized controlled trial[pt] OR controlled clinical trial[pt] OR random*[tiab])) AND ("2014/01/01"[Date - Publication] : "2026/07/09"[Date - Publication])

Scopus

```
TITLE-ABS-KEY(((preterm OR premature OR "very preterm" OR "extremely preterm" OR "very low birth
weight") W/2 (infant* OR neonat*)) AND (physiotherap* OR "physical therap*" OR "motor intervention*"
OR "motor training" OR "sensorimotor intervention*" OR "developmental intervention*" OR "early
intervention*") AND (parent* OR caregiver* OR "family-cent*" OR "parent-delivered" OR "parent-
administered" OR "parent-guided" OR coaching) AND ("motor development" OR neurodevelopment* OR TIMP
OR "general movement*" OR Bayley OR AIMS OR "MOS-R") AND random*) AND PUBYEAR > 2013 AND PUBYEAR <
2027
```

PEDro

```
PEDro Advanced Search was run as a series of field-based searches because Boolean OR is not processed
within PEDro free-text fields. The following combinations were entered in the “Abstract & Title”
field, with Subdiscipline = Paediatrics, Method = Clinical trial, and Published since = 2014: (1)
preterm parent motor; (2) preterm caregiver motor; (3) premature parent physiotherapy; (4) premature
caregiver physiotherapy; (5) preterm family "physical therapy"; and (6) premature family "early
intervention". Results from all runs were exported and deduplicated.
```

Cochrane CENTRAL

```
#1 MeSH descriptor: [Infant, Premature] explode all trees
#2 ((preterm OR premature OR "very preterm" OR "extremely preterm" OR "very low birth weight") NEAR/2
(infant* OR neonat*)):ti,ab,kw
#3 MeSH descriptor: [Physical Therapy Modalities] explode all trees
#4 (physiotherap* OR "physical therap*" OR "motor intervention*" OR "motor training" OR sensorimotor
OR "developmental intervention*" OR "early intervention*"):ti,ab,kw
#5 (parent* OR caregiver* OR "family-cent*" OR "parent-delivered" OR "parent-administered" OR
"parent-guided" OR coaching):ti,ab,kw
```

```
#6 ("motor development" OR neurodevelopment* OR TIMP OR "general movement*" OR Bayley OR AIMS OR
"MOS-R"):ti,ab,kw
#7 (#1 OR #2) AND (#3 OR #4) AND #5 AND #6, limited to Trials and publication years 2014-2026
```

ClinicalTrials.gov

Condition/disease: Preterm Birth OR Premature Infant
Other terms: (physiotherapy OR "physical therapy" OR "motor intervention" OR "motor training" OR sensorimotor OR "developmental intervention") AND (parent OR caregiver OR family)
Study type: Interventional Studies
Age: Child (birth–17 years)
Recruitment status: All
First posted or last updated: through 9 July 2026

Supplementary searching: reference lists of all included reports and recent systematic reviews were screened; forward citation searches were undertaken for each included report and for the NOPPI trial; and trial registrations were checked for linked publications and follow-up reports.

Supplementary Table S2. Full-Text Exclusion Framework and Primary Reasons

Thirty-six full-text reports were assessed in the original audited screening workflow, and 32 were excluded. Each excluded report was assigned one primary reason. The original screening archive supplied for the present revision retained the total number of exclusions and the prespecified reason framework, but it did not retain category-level totals in a form suitable for valid retrospective reconstruction. Accordingly, no category counts have been invented. Linked reports that contributed distinct eligible outcomes were retained, connected to their parent trial, and counted once when estimating independent trials and unique participants.

Primary exclusion reason	Operational definition
Population not eligible	Term-only samples; late-preterm samples (34–36 weeks) without separable data; or mixed high-risk samples without extractable results for infants born before 34 completed weeks.
No active guided parental role	Therapist-delivered or nursing-only interventions; routine developmental advice; or parent contact without structured training, coaching, or guided delivery of a motor/physiotherapy component.
No central physiotherapy or motor component	General developmental, sensory, relational, feeding, music, skin-to-skin, or massage-only programs without a discrete motor-learning, movement-training, or physiotherapy component.
Ineligible design or report type	Non-controlled studies; protocols without outcome data; conference abstracts; qualitative reports; reviews; case series; or secondary analyses that did not report an eligible comparative developmental outcome.
No eligible validated outcome	No validated motor or neurodevelopmental outcome within the prespecified infant follow-up window, or outcomes limited to feasibility, physiology, parent satisfaction, or biomarkers.
Duplicate or linked trial report	Duplicate publications were removed. Companion reports from the same randomized cohort were retained only when they contributed distinct eligible outcomes, linked to one trial identifier, and not double-counted in participant totals.

Screening decision rule. When more than one reason applied, reviewers assigned the first reason encountered in the following hierarchy: population → parental role → intervention focus → design/report type → outcome → duplicate/linked report. Disagreements were resolved by discussion and consensus.

Supplementary Table S3. Standardized Data-Extraction and Appraisal Form

Two reviewers independently extracted study and report data using the following piloted fields. Disagreements were resolved by consensus. The form was designed to support trial-level linkage, TIDieR description, narrative synthesis, RoB 2 assessment, and outcome-level GRADE judgments.

Domain	Data items	Coding / decision rule
Citation and trial identity	Full citation; DOI; registration number; country; recruitment sites and dates; author group; parent trial; linked or companion reports.	Free text; assign one trial identifier to all linked reports.
Study design	Parallel-group RCT/controlled trial; number of arms; allocation method; masking; protocol/SAP availability; funding and conflicts.	Record verbatim and code for RoB 2.
Participants	Randomized and analyzed sample; gestational age; birth weight; sex; medical/neurological risk; inclusion/exclusion criteria; baseline developmental risk.	Report by group and for the total randomized cohort.
Timing and setting	Postmenstrual/corrected age at enrolment; NICU, hospital, outpatient, home, community, or telehealth setting; duration and follow-up time points.	Use corrected age consistently where applicable.
Intervention rationale	Theoretical basis; intended mechanism; motor-learning principles; family-centered or coaching framework.	Map to TIDieR rationale/why.
Intervention materials and procedures	Written/video materials; equipment; positioning; handling; tactile input; assisted movement; active exploration; play; home activities; progression rules.	Separate passive, assisted, and infant-initiated active elements.
Provider and parent training	Professional discipline; number and content of training sessions; demonstration; supervised practice; teach-back; cue recognition; stopping criteria; follow-up support.	Describe both professional and parent roles.
Dose and tailoring	Frequency; session duration; program duration; intended and received dose; individualization; progression; booster contacts.	Extract planned and achieved dose separately.
Comparator	Usual care, developmental care, active physiotherapy, no intervention; co-interventions; contamination; access to follow-up services.	Describe comparator content, not only label.

Domain	Data items	Coding / decision rule
Outcomes	Instrument; developmental domain; assessor; assessor masking; corrected-age time point; primary/secondary status; clinical threshold; normative reference.	Extract all eligible time points and distinguish parent-reported from performance-based measures.
Effect data	Group means/SDs, counts, adjusted estimates, mean differences, standardized effects, confidence intervals, p values, and direction of effect as reported.	No effect estimate was reconstructed when necessary statistics were unavailable.
Implementation	Adherence; fidelity; reasons for missed sessions; acceptability; feasibility; continuation over time; family burden; socioeconomic/language barriers.	Record measurement method and time course.
Safety	Adverse events; overstimulation or intolerance; infant stress/disengagement cues; stop criteria; intrusive handling; supervision and escalation procedures.	Record explicit absence of events only when reported.
Attrition and missing data	Numbers lost by time point; reasons; differential loss; analysis population; sensitivity analyses; handling of missing outcomes.	Inform RoB 2 domain 3.
RoB 2 and GRADE	Domain-level signaling information and rationale; overall RoB 2 judgment; GRADE downgrading for risk of bias, inconsistency, indirectness, imprecision, and publication bias.	Assess independently, reconcile by consensus, and document rationale.

Supplementary Table S4. Detailed Cochrane RoB 2 Supporting Judgments

Two reviewers independently applied RoB 2 [22] to a prespecified principal motor or developmental result from each independent randomized cohort and reconciled disagreements by consensus. A formal agreement coefficient was not prespecified or calculated. Judgments reflect the effect of assignment to intervention and are based on information available in the published reports and associated registrations. D1, randomization process; D2, deviations from intended interventions; D3, missing outcome data; D4, measurement of the outcome; D5, selection of the reported result.

Report	Target result(s) assessed
NOPPI cohort: Ustad et al. (2016) [25] and Fjørtoft et al. (2017) [26]	Principal short-term TIMP result at 37 weeks postmenstrual age and linked GMA/movement-character result at 3 months corrected age
Kara et al. (2019) [30]	Bayley-III fine- and gross-motor outcomes through the final reported follow-up
Ochandorena-Acha et al. (2022) [31]	AIMS and ASQ-3 developmental outcomes at the later reported follow-up
Fucile et al. (2025) [32]	TIMP motor outcome in the neonatal parent-administered sensorimotor intervention trial
Badura et al. (2026) [21]	MOS-R at 3–5 months and Bayley-III neurodevelopment at 18–24 months corrected age

Report	Domain	Judgment	Supporting rationale
NOPPI cohort [25,26]	D1 Randomization	Low risk	The parent trial reported randomized allocation, with no clear evidence of a compromised sequence or important baseline imbalance.
NOPPI cohort [25,26]	D2 Deviations	Low risk	Parents and therapists could not be blinded, but intervention delivery was trained and supervised and no important differential deviations likely to invalidate the assignment effect were reported.
NOPPI cohort [25,26]	D3 Missing data	Some concerns	The short-term TIMP endpoint was available for most randomized infants, whereas the linked 3-month GMA analysis included 130 of 153 infants; the influence of missing follow-up data could not be ruled out completely.
NOPPI cohort [25,26]	D4 Measurement	Low risk	TIMP and General Movement Assessment are standardized measures, and outcome assessment was reported as masked to group assignment.
NOPPI cohort [25,26]	D5 Reported result	Some concerns	The trial was registered, but a fully accessible prespecified statistical analysis plan for all linked outcomes was not available; selective result reporting cannot be excluded.
NOPPI cohort [25,26]	Overall	Some concerns	The overall judgment was driven by incomplete follow-up for the linked GMA result and uncertainty about selection of reported results.
Kara 2019 [30]	D1 Randomization	Low risk	Random allocation was reported and no important baseline imbalance suggesting a failed randomization process was evident.

Report	Domain	Judgment	Supporting rationale
Kara 2019 [30]	D2 Deviations	Low risk	Both groups received structured intervention, and no clear differential deviation or contamination likely to invalidate the assignment comparison was reported.
Kara 2019 [30]	D3 Missing data	Some concerns	The sample was small and attrition accumulated over longer follow-up without a robust sensitivity analysis for missing outcomes.
Kara 2019 [30]	D4 Measurement	Low risk	Bayley-III is a validated standardized measure and assessor blinding was reported.
Kara 2019 [30]	D5 Reported result	Some concerns	A prespecified statistical analysis plan was not available, so selective reporting across repeated time points cannot be excluded.
Kara 2019 [30]	Overall	Some concerns	The overall judgment was driven by attrition, small sample size, and uncertainty about selective reporting.
Ochandorena-Acha 2022 [31]	D1 Randomization	Low risk	A randomized, single-blind design was reported with no clear evidence of compromised allocation or important baseline imbalance.
Ochandorena-Acha 2022 [31]	D2 Deviations	Low risk	Participant and provider masking was not feasible, but no important differential deviations from intended care were identified; adherence was incompletely described.
Ochandorena-Acha 2022 [31]	D3 Missing data	Low risk	Retention for the principal follow-up outcomes was adequate and missingness was unlikely to overturn the comparison.
Ochandorena-Acha 2022 [31]	D4 Measurement	Low risk	Validated AIMS and ASQ-3 outcomes were used and the single-blind design reduced concern about assessor knowledge of allocation.
Ochandorena-Acha 2022 [31]	D5 Reported result	Some concerns	The available reporting did not provide a fully documented prespecified analysis plan for all outcomes and time points.
Ochandorena-Acha 2022 [31]	Overall	Some concerns	The overall judgment was driven by uncertainty in selection of the reported result.
Fucile 2025 [32]	D1 Randomization	Some concerns	A randomized design was reported, but publicly available detail on sequence generation and allocation concealment was limited.
Fucile 2025 [32]	D2 Deviations	Low risk	The intervention was standardized, parent delivery was monitored with explicit stopping rules, and no important differential deviations from assigned care were evident.
Fucile 2025 [32]	D3 Missing data	Some concerns	Eighty of 94 randomized infants completed the neonatal study, so the influence of missing outcome data could not be excluded completely.
Fucile 2025 [32]	D4 Measurement	Low risk	TIMP is a validated standardized motor measure, and there was no indication that outcome measurement differed systematically between groups.

Report	Domain	Judgment	Supporting rationale
Fucile 2025 [32]	D5 Reported result	Some concerns	A fully accessible prespecified statistical analysis plan covering all outcomes and time points was not available; selective reporting remains possible.
Fucile 2025 [32]	Overall	Some concerns	The overall judgment was driven by limited randomization detail, incomplete outcome data, and uncertainty about result selection.
Badura 2026 [21]	D1 Randomization	Some concerns	An independent randomization list was used, but twins were assigned to the same group after randomization, creating some concern about the randomization process.
Badura 2026 [21]	D2 Deviations	Low risk	Parents and therapists were unblinded and adherence varied, but no important differential deviations or co-interventions likely to invalidate the effect of assignment were identified.
Badura 2026 [21]	D3 Missing data	Some concerns	Long-term follow-up completeness and handling of missing neurodevelopmental data raised concern in a modest sample.
Badura 2026 [21]	D4 Measurement	Low risk	MOS-R and Bayley-III are standardized measures and blinded outcome assessment reduced concern for differential measurement.
Badura 2026 [21]	D5 Reported result	Some concerns	Prespecification and the statistical analysis plan were only partly documented in the accessible report and registration materials.
Badura 2026 [21]	Overall	Some concerns	The overall judgment was driven by the randomization concern, missing follow-up data, and incomplete prespecification detail.

Supplementary Table S5. PRISMA 2020 Checklist and Location Map

The checklist wording is adapted from the PRISMA 2020 checklist [20] under the Creative Commons Attribution 4.0 licence. Page references correspond to the corrected manuscript supplied with this supplementary file; section and table references remain valid if pagination changes during typesetting.

Section / topic	Item	PRISMA 2020 requirement	Location in revised manuscript
Title	1	Identify the report as a systematic review.	Title, p. 1.
Abstract	2	Report a structured summary consistent with PRISMA 2020 for Abstracts.	Abstract, pp. 1–2.
Rationale	3	Describe the rationale for the review in the context of existing knowledge.	Introduction, pp. 3–4.
Objectives	4	Provide an explicit statement of the objective(s) or question(s).	Introduction, final paragraph, p. 4.
Eligibility criteria	5	Specify inclusion and exclusion criteria and how studies were grouped for synthesis.	Section 2.2, pp. 4–5; Section 2.6, p. 7.
Information sources	6	Specify all databases, registers, websites, reference lists, and other sources, and the date each was last searched.	Section 2.3, pp. 5–6; Supplementary Table S1.
Search strategy	7	Present the full search strategies for all databases, registers, and websites, including limits and filters.	Supplementary Table S1.

Section / topic	Item	PRISMA 2020 requirement	Location in revised manuscript
Selection process	8	Specify methods used to decide whether studies met eligibility criteria, including reviewer numbers and independence.	Section 2.4, p. 6.
Data collection process	9	Specify methods used to collect data, including reviewer numbers, independence, and investigator contact if applicable.	Section 2.4, p. 6; Supplementary Table S3.
Data items	10a	List and define all outcomes sought and how results/time points were selected.	Section 2.2, pp. 4–5; Section 2.6, p. 7; Supplementary Table S3.
Data items	10b	List and define all other variables sought and any assumptions about missing or unclear information.	Section 2.4, p. 6; Supplementary Table S3.
Risk of bias	11	Specify the methods and tool used to assess risk of bias, reviewer procedures, and independence.	Section 2.5, pp. 6–7; Supplementary Table S4.
Effect measures	12	Specify the effect measures used for each outcome.	Section 2.6, p. 7; Table 3, pp. 17–18; Supplementary Table S3.
Synthesis methods	13a	Describe how studies were judged eligible for each synthesis.	Sections 2.2 and 2.6, pp. 4–7.
Synthesis methods	13b	Describe data preparation methods, including missing statistics and conversions.	No quantitative pooling or conversion was undertaken; Section 2.6, p. 7; Supplementary Table S3.
Synthesis methods	13c	Describe methods used to tabulate or visually display results.	Tables 1–4 and Figures 1–2, pp. 9–18.
Synthesis methods	13d	Describe synthesis methods and rationale; report meta-analysis models/software if used.	Narrative synthesis and rationale, Section 2.6, p. 7; no meta-analysis.
Synthesis methods	13e	Describe methods used to explore possible causes of heterogeneity.	Narrative comparison of intervention, comparator, dose, outcomes, and follow-up; Sections 2.6 and 4.3–4.5, pp. 7 and 20–22.
Synthesis methods	13f	Describe sensitivity analyses.	Not conducted because no meta-analysis was performed and the evidence base was small; Section 2.6, p. 7.
Reporting bias	14	Describe methods used to assess bias due to missing results.	Formal funnel-plot/small-study methods were not appropriate; publication bias was considered qualitatively in GRADE and Section 4.7, p. 22.
Certainty assessment	15	Describe methods used to assess certainty/confidence in the body of evidence.	Section 2.5, pp. 6–7; Table 4, p. 18.
Study selection	16a	Describe search and selection results from identification to included studies, ideally with a flow diagram.	Section 3.1, p. 8; Figure 1, pp. 9–10.

Section / topic	Item	PRISMA 2020 requirement	Location in revised manuscript
Study selection	16b	Cite studies that might appear eligible but were excluded, and explain why.	Supplementary Table S2; exclusion categories summarized in Figure 1, p. 9.
Study characteristics	17	Cite each included study and present its characteristics.	Section 3.2, p. 8; Table 1, p. 11.
Risk of bias in studies	18	Present risk-of-bias assessments for each included study/report.	Section 3.4, pp. 13–14; Figure 2, p. 14; Supplementary Table S4.
Results of individual studies	19	For each study and outcome, present group data and an effect estimate with precision where available.	Section 3.5, pp. 15–18; Table 3, pp. 17–18; estimates reported as available in source reports.
Results of syntheses	20a	Summarize characteristics and risk of bias among contributing studies for each synthesis.	Sections 3.2–3.6 and Tables 1–4, pp. 8–18.
Results of syntheses	20b	Present results of all statistical syntheses.	No meta-analysis; narrative results in Section 3.5 and Table 3, pp. 15–18.
Results of syntheses	20c	Present results of investigations of heterogeneity.	Narrative interpretation in Sections 4.3–4.5, pp. 20–22.
Results of syntheses	20d	Present results of sensitivity analyses.	Not applicable; no sensitivity analysis was conducted.
Reporting biases	21	Present assessments of bias due to missing results for each synthesis.	No formal assessment was possible because of the small number of independent cohorts; limitation discussed in Section 4.7, p. 22.
Certainty of evidence	22	Present certainty/confidence assessments for each outcome.	Section 3.6 and Table 4, p. 18.
Discussion	23a	Provide a general interpretation of results in the context of other evidence.	Sections 4.1–4.5, pp. 19–22.
Discussion	23b	Discuss limitations of the included evidence.	Section 4.7, p. 22.
Discussion	23c	Discuss limitations of the review processes.	Section 4.7, p. 22.
Discussion	23d	Discuss implications for practice, policy, and future research.	Sections 4.6 and 4.8 and Conclusions, pp. 22–23.
Registration/protocol	24a	Provide registration information, including register and registration number.	Section 2.1, p. 4; PROSPERO identifier 1137282.

Section / topic	Item	PRISMA 2020 requirement	Location in revised manuscript
Registration/protocol	24b	Indicate where the protocol can be accessed, or state that no protocol was prepared.	Section 2.1, p. 4; PROSPERO record 1137282.
Registration/protocol	24c	Describe and explain amendments to registration/protocol information.	Revision-stage source expansion and search update described in Sections 2.1–2.3, pp. 4–6, and Supplementary Table S1.
Support	25	Describe financial and non-financial support and the role of funders/sponsors.	Funding statement, p. 23.
Competing interests	26	Declare competing interests of review authors.	Conflicts of Interest statement, p. 23.
Availability of materials	27	Report availability of data-collection forms, extracted data, analysis data/code, and other review materials.	Data Availability Statement, p. 23; Supplementary Tables S1–S4; study-level extracted data summarized in Tables 1–4.

Supplementary References

20. Page, M.J.; McKenzie, J.E.; Bossuyt, P.M.; Boutron, I.; Hoffmann, T.C.; Mulrow, C.D.; Shamseer, L.; Tetzlaff, J.M.; Akl, E.A.; Brennan, S.E.; et al. The PRISMA 2020 statement: An updated guideline for reporting systematic reviews. BMJ 2021, 372, n71. https://doi.org/10.1136/bmj.n71.

21. Badura, A.; Dietz, A.; Zeman, F.; Klarner, S.; Ammon, L.; Waltner-Romen, M.; Griesmaier, E.; Wellmann, M.; Lehnerer, V.; Wellmann, S. General movement based therapy to support neurodevelopment of preterm infants: A randomized clinical trial. Pediatr. Res. 2026. https://doi.org/10.1038/s41390-025-04734-2.

22. Sterne, J.A.C.; Savović, J.; Page, M.J.; Elbers, R.G.; Blencowe, N.S.; Boutron, I.; Cates, C.J.; Cheng, H.Y.; Corbett, M.S.; Eldridge, S.M.; et al. RoB 2: A revised tool for assessing risk of bias in randomised trials. BMJ 2019, 366, l4898. https://doi.org/10.1136/bmj.l4898.

25. Ustad, T.; Evensen, K.A.I.; Campbell, S.K.; Girolami, G.L.; Helbostad, J.; Jørgensen, L.; Kaaresen, P.I.; Øberg, G.K. Early parent-administered physical therapy for preterm infants: A randomized controlled trial. Pediatrics 2016, 138, e20160271. https://doi.org/10.1542/peds.2016-0271.

26. Fjørtoft, T.; Ustad, T.; Follestad, T.; Kaaresen, P.I.; Øberg, G.K.; Labori, C.; Støen, R. Does a parent-administrated early motor intervention influence general movements and movement character at 3 months of age in infants born preterm? Early Hum. Dev. 2017, 112, 20–24. https://doi.org/10.1016/j.earlhumdev.2017.06.008.

30. Kara, O.K.; Sahin, S.; Yardimci-Lokmanoglu, B.N.; Mutlu, A. The role of the family in early intervention of preterm infants with abnormal general movements. Neurosciences 2019, 24, 101–109. https://doi.org/10.17712/nsj.2019.2.20180001.

31. Ochandorena-Acha, M.; Terradas-Monllor, M.; López Sala, L.; Cazorla Sánchez, M.E.; Fornaguera Martí, M.; Muñoz Pérez, I.; Agut-Quijano, T.; Iriondo, M.; Casas-Baroy, J.C. Early physiotherapy intervention program for preterm infants and parents: A randomized, single-blind clinical trial. Children 2022, 9, 895. https://doi.org/10.3390/children9060895.

32. Fucile, S.; Snider, L.; O'Brien, K.; Smith, L.; Dow, K. A parent-administered sensorimotor intervention for oral feeding in infants born preterm: A randomized clinical study. Dev. Med. Child Neurol. 2025, 67, 208–215. https://doi.org/10.1111/dmcn.16046.

PRISMA checklist attribution: Page et al. (2021) [20]; checklist content used under CC BY 4.0.
